# *In Vivo* Biocompatibility Study of Electrospun Chitosan Microfiber for Tissue Engineering

**DOI:** 10.3390/ijms11104140

**Published:** 2010-10-25

**Authors:** Yun Mi Kang, Bit Na Lee, Jae Hoon Ko, Gyeong Hae Kim, Kkot Nim Kang, Da Yeon Kim, Jae Ho Kim, Young Hwan Park, Heung Jae Chun, Chun Ho Kim, Moon Suk Kim

**Affiliations:** 1 Department of Molecular Science and Technology, Ajou University, Suwon, 443-749, Korea; E-Mails: ymkang@ajou.ac.kr (Y.M.K.); leeanm87@ajou.ac.kr (B.N.L.); redboykh@ajou.ac.kr (G.H.K.); knkang@ajou.ac.kr (K.N.K.); dayeon@ajou.ac.kr (D.Y.K.); jhkim@ajou.ac.kr (J.H.K.); 2 Digital Dyeing & Finishing Tech, Korea Institute of Industrial Technology, Ansan, 426-791, Korea; E-Mails: ellafiz@kitech.re.kr (J.H.K.); yhpark@kitech.re.kr (Y.H.P.); 3 Department of Biomedical Sciences, College of Medicine, Catholic University, Seoul, 137-701, Korea; E-Mail: chunhj@catholic.ac.kr; 4 Laboratory of Tissue Engineering, Korea Institute of Radiological and Medical Sciences, Seoul, 139-706, Korea; E-Mail: chkim@kcch.re.kr

**Keywords:** electrospun, chitosan, microfibers, scaffold, stem cell

## Abstract

In this work, we examined the biocompatibility of electrospun chitosan microfibers as a scaffold. The chitosan microfibers showed a three-dimensional pore structure by SEM. The chitosan microfibers supported attachment and viability of rat muscle-derived stem cells (rMDSCs). Subcutaneous implantation of the chitosan microfibers demonstrated that implantation of rMDSCs containing chitosan microfibers induced lower host tissue responses with decreased macrophage accumulation than did the chitosan microfibers alone, probably due to the immunosuppression of the transplanted rMDSCs. Our results collectively show that chitosan microfibers could serve as a biocompatible *in vivo* scaffold for rMDSCs in rats.

## 1. Introduction

Tissue engineering has developed as a way to repair and regenerate defective or damaged tissues and organs [1]. The typical method is to incorporate various cells into three-dimensional polymer scaffolds and to create conditions for the cells to proliferate *in vitro* and *in vivo*. The polymer scaffold controls the tissue structure by holding the cells together in a particular three-dimensional structure and by regulating their biological function [2].

Recently, developing scaffolds that mimic the architecture of three-dimensional tissue at the micro/nanoscale is one of the major challenges in the field of tissue engineering [3,4]. The three-dimensional scaffolds at the micro/nanoscale may serve as an excellent framework for the attachment, growth, and differentiation of implanted cells. The three-dimensional scaffolds may also provide a suitable environment for the diffusion of nutrients, metabolites and biological factors. Such a scaffold, therefore, needs to be developed to mimic the structure and biological functions of extracellular matrices (ECM) [5].

Currently, there are three techniques available for the fabrication of micro/nanoscale scaffolds: electrospinning, self-assembly, and phase separation [6–8]. Of these techniques, electrospinning is considered as one of the most promising techniques. Therefore, the development of micro/nanoscale scaffolds by using electrospinning has greatly enhanced the scope for fabricating scaffolds that can fulfill various challenges in terms of tissue engineering applications. The availability of a wide range of natural and synthetic biomaterials has broadened the scope for development of micro/nanoscale scaffolds [9,10]. Among them, chitosan-based scaffolds have been shown to be biodegradable, non-immunogenic and biocompatible, and thus are widely used as therapeutic scaffolds for tissue engineering processes [11–13].

Adult stem cells are self-renewing and pluripotent cells with a plasticity to differentiate into cell types of a particular tissue [14]. These adult stem cells have been obtained from various sources such as bone marrow, adipose tissue, muscle tissue, and human umbilical cord. Because of its considerable mass in the body, skeletal muscle has attracted much attention as a potential source of adult stem cells. Muscle-derived stem cells (MDSCs) can be isolated from skeletal muscles and have been shown to undergo multilineage differentiation *in vitro* and *in vivo* upon stimulation with several biological factors [15–17]. Here, we examined the ability of microscale scaffolds prepared by electrospinning of chitosan to create a suitable *in vitro* and *in vivo* substrate for rat MDSCs (rMDSCs). In addition, we evaluated *in vivo* host tissue responses to rMDSCs-containing chitosan microfibers.

## 2. Experimental Section

### 2.1. Preparation of Electronspun Chitosan Microfibers

Chitosan solution (5 wt%) was prepared by dissolving the chitosan (*M*_w_ = 50,000, degree of deacetylation = 97%) in 2 wt% CH_3_COOH solution. Typically, electrospinning was performed at 20 kV voltage, 10 cm distance between the needle tip and the collector (Taksan Meditech Co., Ltd., Korea). The flow rate of the solution was controlled by a syringe pump maintained at 10 m/min from the needle outlet. A grounded aluminum foil was used as the collector. The microfibrous chitosan was collected on the surface of aluminum foil. A porosimeter (Auto Pore IV 9500 V 1.03; Instruments Co., USA) was used to examine the porosity and mean pore diameter of the obtained microfibrous chitosan.

### 2.2. Rat Muscle-Derived Stem Cell Isolation

The major hind-limb muscles of the rats were removed, trimmed of excess connective tissue and fat, hand minced, and washed twice with PBS. After centrifugation at 2000 rpm for 5 min, the resultant pellets were enzymatically dissociated by adding 0.2% collagenase-type XI (Sigma, Germany) for 1 h at 37 °C, dispase (20 mL, Gibco BRL) for 45 min, and 0.1% trypsin (Sigma, Germany) for 30 min. Cells were separated from muscle fiber fragments and tissue debris by differential centrifugation and were plated on collagen-coated flasks in DMEM containing 5% fetal bovine serum (Gibco BRL), 5% horse serum (Gibco BRL), penicillin (100 U/mL), and streptomycin (100 μg/mL) for 1 h. The muscle cell extract was pre-plated on collagen-coated flasks. Unattached cells floating in the medium after 1 h incubation were then transferred onto fresh collagen-coated flasks. The subsequent pre-plates were performed in the next 2 h, 3 h, 1 day, 2 days and 3 days. Finally, the rMDSCs were seeded into normal tissue culture flasks at 1 × 10^5^ cells/cm^2^. The flasks were rinsed three times with PBS on the second day of expansion. The medium was changed every 2 days throughout the studies. Adherent cells, rMDSCs, were rinsed thoroughly with PBS and detached by 0.05% trypsin-EDTA for experiment use. The final rMDSCs used were taken at passage 5.

### 2.3. PKH67 Cell Labeling

The rMDSCs were labeled using the PKH67 Fluorescent Cell Linker Kit (Sigma, USA) according to the manufacturer’s instructions. In brief, the cultured rMDSCs were washed with serum-free media and centrifuged for 5 min at 400 g. The provided diluent C (500 μL) was added to 3 × 10^5^ rMDSCs and immediately mixed with 500 μL of PKH67 stock solution (4 × 10^−6^ M) in diluent C. After incubation for 5 min at room temperature, 1 mL of FBS was added and samples were incubated for 1 min to stop the labeling reaction. Finally, the rMDSCs were pelleted for 5 min at 400 g, transferred to a fresh tube and washed three times with complete DMEM.

### 2.4. Cell Culture on Electronspun Chitosan Microfibers

Electronspun chitosan microfibers were sterilized by EO gas at Hansbiomed Company. For cell culture experiments, the chitosan microfibers were prepared and placed individually into the wells of a 24-well tissue culture plate (Falcon, USA) and then incubated for 1 h in culture media. After suction of the media, the PKH67 labeled rMDSCs (3 × 10^4^ cells/well) were transferred to each well. The culture media was changed every 2 days throughout the studies. For SEM measurements, at 1 and 7 days, the microfibrous scaffolds without or with rMDSCs were fixed with 2.5% glutaraldehyde for 24 h, followed by ethanol dehydration. The fixed chitosan microfibers were coated with a conductive layer of gold using a plasma-sputtering apparatus (Emitech, K575, Kent, UK), and scanning electron microscopy (SEM, S-2250N, Hitachi, Japan) images were obtained. Cytotoxicity was measured using a WST-1 kit (Roche, Germany) after 1, 3, and 7 days. Briefly, 100 μL of WST-1 reagent was added to rMDSCs in 1 mL medium per well, the plates were incubated at 37 °C for 4 h, and the samples were then shaken for 1 min. An aliquot from each well (100 μL) was transferred to a 96-well plate, and absorbance at 450 nm was measured with a microplate reader (EL808 ultra microplate reader; Bio-Tek Instrument, USA).

### 2.5. Animal Implantation Surgery

The rats were housed in sterilized cages with sterile food, water and filtered air, and were handled under a laminar flow hood following aseptic techniques. All animals were treated in accordance with the Institutional Animal Experiment Committee at Ajou University School of Medicine. Twenty-four Fischer rats (140–160 g, 4 weeks), divided into four groups of three rats each, were used in the animal tests. The chitosan microfibers without and with rMDSCs (3 × 10^4^ cells) were implanted subcutaneously under the dorsal skin and then allowed to develop, and biopsied *in vivo* over 4 weeks. At each of the post-implantation points, the rats were sacrificed and the implants were dissected individually and removed from the subcutaneous dorsum.

### 2.6. *In Vivo* SEM Measurements

SEM was used to examine morphology of *in vivo* chitosan microfibers. The *in vivo* chitosan microfibers without or with rMDSCs after 1 and 4 weeks were fixed with 2.5% glutaraldehyde for 24 h, followed by ethanol dehydration. The fixed chitosan microfibers were coated with a conductive layer of gold using a plasma-sputtering apparatus (Emitech, K575, Kent, UK), and scanning electron microscopy (SEM, S-2250N, Hitachi, Japan) images were obtained.

### 2.7. Histological Analysis

At 3 days, one, two and four weeks after implantation, the rats were sacrificed and the implants were individually dissected and removed from the subcutaneous dorsum. The implants were immediately fixed with 10% formalin and embedded in paraffin. The embedded specimens were sectioned (4 μm) along the longitudinal axis of the implant, and the sections were stained with hematoxylin and eosin (H&E), 6-diamino-2-phenylindoadihydrochloride (DAPI, Sigma-Aldrich, St Louis, MO, USA), and mouse anti-rat CD68 (ED1; 1:1000; Serotec, Oxford, UK). The staining procedures for DAPI and ED1 were as follows. The slides were washed with PBS-T (0.05% Tween 20 in PBS), blocked with 5% bovine serum albumin (BSA; Roche, Mannheim, Germany) and 5% horse serum (HS; GIBCO, Paisley, UK) in PBS for 1 h at 37 °C. The sections were incubated overnight at 4 °C with ED1 antibodies, washed with PBS-T, and then incubated with the secondary antibody (goat anti-mouse Alexa Fluor^®^594; Invitrogen, San. Diego, CA, USA) for 3 h at room temperature in the dark. The slides were washed again with PBS-T, counterstained with DAPI, and then mounted with fluorescent mounting solution (DAKO, Calif, USA). Immunofluorescent images were visualized under an Axio Imager A1 (Carl Zeiss Microimaging GmbH, Göttingen, Germany) equipped with Axiovision Rel. 4.8 software (Carl Zeiss Microimaging GmbH, Göttingen, Germany). Before acquisition of immunofluorescent images, the delimitation between implant and host tissue was determined from a differential interference contrast (DIC) optical microscopic image. The sections were also stained with hematoxylin and eosin (H&E) using standard procedures.

### 2.8. Statistical Analysis

Cytotoxicity data were obtained from independent experiments in which three wells per culture plate or chitosan microfibers were examined and ED-1 assays were carried out in independent experiments with *n* = 16 for each data point, with data given as the mean and standard deviation (SD). Statistical analysis using the one-way ANOVA method was carried out with Bonferroni’s multiple comparisons.

## 3. Results and Discussion

**Figure 1:**
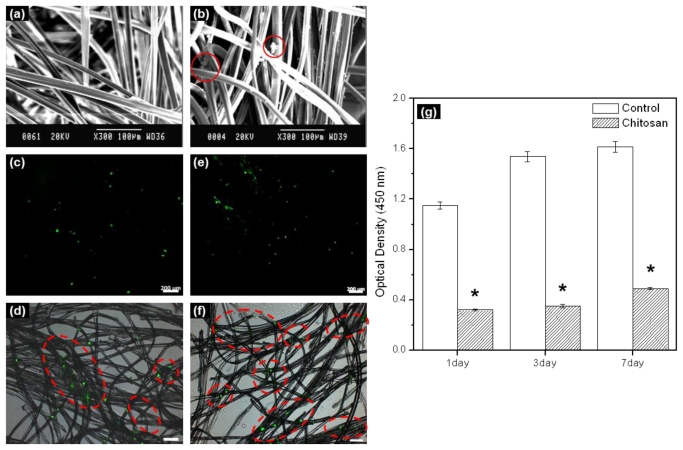
SEM images of (**a**) chitosan microfiber only, (**b**) rMDSCs seeded on chitosan microfiber at 1 day, and fluorescence images of PHK67 labeled rMDSCs seeded on chitosan microfiber at (**c**, **d**) 1 and (**e**, **f**) 7 days. Magnification: (**a**, **b**) 300 x, (**c**, **e**) 50 x and (**d**, **f**) 100 x. Scale bars: (**a**, **b**) 100 μm, (**c**, **e**) 200 μm and (**d**, **f**) 100 μm. rMDSCs viability on chitosan microfiber measured by WST-1 assay (**g**). rMDSCs grown on a plain culture plate were used as the control (* *p* < 0.005).

In this work, electrospun chitosan microfibers exhibited randomly arranged structures. Figure 1a shows a SEM image. The electrospun chitosan microfiber had an average diameter of ~10 μm, pore diameter of 22 μm, and porosity of 90%. Adequate three-dimensional structure and porosity is required not only to achieve sufficient cell seeding conditions within the chitosan microfibers as a scaffold, but also to facilitate cell proliferation and differentiation by allowing the transport of nutrients and oxygen into and out of the chitosan microfiber scaffold.

In order to examine the rMDSCs attaching and proliferation on the chitosan microfibers after 1 and 7 days, rMDSCs attaching and proliferation on the chitosan microfibers were examined by SEM and fluorescence microscopy. SEM images showed the morphology of the rMDSCs attaching on the chitosan microfibers after 1 day (Figure 1b). The fluorescence image of PHK67 labeled rMDSCs was observed on the chitosan microfibers (Figure 1c–f). The number of green images increased with increasing incubation time. The green fluorescence images mean the PHK67 labeled rMDSCs.

We evaluated the cytotoxicity of *in vitro* cultured rMDSCs on chitosan microfibers over a period of 7 days (Figure 1g). For comparison, identical experiments were carried out on fibers-free tissue culture plates. The number of rMDSCs generally increased on both the chitosan microfibers and the tissue culture plate (control) (* *p* < 0.005). Therefore, we found that chitosan microfibers were biocompatible substrates for the attachment and proliferation of rMDSCs, even though significantly more rMDSCs were counted on the tissue culture plate compared to the chitosan microfibers on 1, 3, and 7 days.

**Figure 2:**
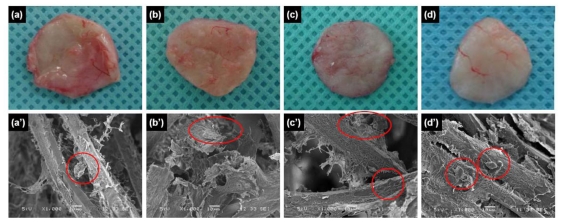
Optical (upper) and SEM (bottom) images of chitosan microfibers removed from rats after (**a**, **a**′) 3 days, (**b**, **b**′) 1 week, (**c**, **c**′) 2 weeks, and (**d**, **d**′) 4 weeks.

To examine the utility of chitosan microfibers as *in vivo* scaffolds, we implanted chitosan microfibers without and with rMDSCs subcutaneously into rats. The implants were allowed to develop for up to four weeks *in vivo*, and were excised and examined at various times post-implantation. The resulting implants maintained their shapes even after four weeks *in vivo* (Figure 2a–d). In addition, vascular blood vessels formed around the surfaces of the implants over time after implantation. SEM images showed chitosan microfibers interspersed with connective tissues and cells (Figure 2a′–d′).

**Figure 3:**
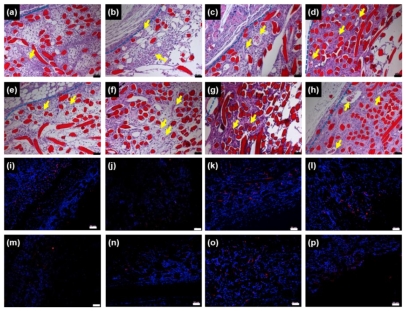
H&E (**a**–**h**) and ED1 immunofluorescence (**i**–**p**) staining of chitosan microfibers (**a**–**d**, **i**–**l**) without and (**e**–**h**, **m**–**p**) with rMDSCs removed from rats after (**a**, **e**, **i**, **m**) 3 days, (**b**, **f**, **j**, **n**) 1 week, (**c**, **g**, **k**, **o**) 2 weeks, and (**d**, **h**, **l**, **p**) 4 weeks. Scale bar is 50 μm.

To observe *in vivo* chitosan microfibers as a scaffold in more detail, we performed histological staining of tissue within and near the implanted chitosan microfibers of rats 3 days, 1, 2 and 4 weeks post-implantation. H&E staining of chitosan microfibers (red) could be clearly distinguished from the host tissue layer. New blood vessels were observed in chitosan microfibers, in which the arrows indicate newly formed blood vessels (Figure 3a–h, upper).

**Figure 4:**
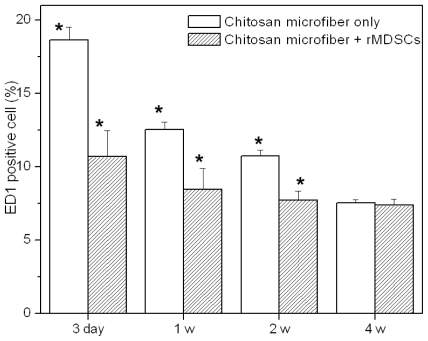
The number of ED1-positive cells on chitosan microfibers at 3 day, 1 week, 2 weeks and 4 weeks post implantation (* *p* < 0.05).

The extent of host cell infiltration and inflammatory cell accumulation within and surrounding the chitosan microfibers was also characterized by staining tissue with ED1 (red) to identify monocytes/macrophages; nuclei were stained with DAPI (blue) (Figure 3i–p, bottom). DAPI staining revealed the presence of numerous host cells within and surrounding the chitosan microfibers. ED1 staining showed that macrophages were present in chitosan microfibers. The ED1-positive cells were counted and normalized to the total stained tissue area to determine the extent of inflammation (Figure 4). The number of macrophages (ED1-positive cells) decreased slightly with increasing implant duration (*p < 0.05). The rats receiving rMDSCs-containing chitosan microfibers showed slightly less macrophages than chitosan nanofiber only, due to the unique immunomodulatory properties of stem cells [18–20]. In addition, the extent of inflammation of chitosan microfibers with and without rMDSCs was less pronounced than that to the FDA-approved poly(lactic-co-glycolic acid) (PLGA) [21,22].

## 4. Conclusions

We herein prepared chitosan microfibers as a scaffold. It has a three-dimensional pore structure to support attachment and viability of rMDSCs. The implantation of chitosan microfibers containing rMDSCs induced lower host tissue responses than did the chitosan microfibers alone. This result showed that chitosan microfibers could be used as a biocompatible *in vivo* scaffold for rMDSCs in rats. Further research on the animal model with body defect for comparing biocompatibility of chitosan microfibers prepared in this work will be reported on in the future.
